# Biosensors; nanomaterial-based methods in diagnosing of *Mycobacterium tuberculosis*

**DOI:** 10.1016/j.jctube.2023.100412

**Published:** 2023-12-25

**Authors:** Ahmad Mobed, Mohammad Darvishi, Fereshteh Kohansal, Fatemeh Moradi Dehfooli, Iraj Alipourfard, Amir Tahavvori, Farhood Ghazi

**Affiliations:** a Infectious and Tropical Diseases Research Center, Clinical Research Institute, Tabriz University of Medical Sciences, Iran; b Infectious Diseases and Tropical Medicine Research Center (IDTMRC), Department of Aerospace and Subaquatic Medicine, AJA University of Medical Sciences, Tehran, Iran; c Drug Applied Research Center, Tabriz University of Medical Sciences, Tabriz, Iran; d Institute of Medical Science and Technology, Shahid Beheshti University, Tehran, Iran; e Institute of Medical Science and Technology, Tehran University of Medical Sciences, Tehran, Iran; f Internal Department, Medical Faculty, Urmia University of Medical Sciences, Iran

**Keywords:** Pulmonary infection, *Mycobacterium tuberculosis* (Mtb), Conventional methods, Biosensors, Sensitivity

## Abstract

Diagnosis of *Mycobacterium tuberculosis (Mtb)* before the progression of pulmonary infection can be very effective in its early treatment. The Mtb grows so slowly that it takes about 6–8 weeks to be diagnosed even using sensitive cell culture methods. The main opponent in tuberculosis (TB) and nontuberculous mycobacterial (NTM) epidemiology, like in all contagious diseases, is to pinpoint the source of infection and reveal its transmission and dispersion ways in the environment. It is crucial to be able to distinguish and monitor specific *mycobacterium* strains in order to do this. In food analysis, clinical diagnosis, environmental monitoring, and bioprocess, biosensing technologies have been improved to manage and detect Mtb. Biosensors are progressively being considered pioneering tools for point-of-care diagnostics in Mtb discoveries. In this review, we present an epitome of recent developments of biosensing technologies for *M. tuberculosis* detection, which are categorized on the basis of types of electrochemical, Fluorescent, Photo-thermal, Lateral Flow, Magneto-resistive, Laser, Plasmonic, and Optic biosensors.

## 1 Introduction

There are more than 140 species of Mycobacterium which are divided into three groups: *M. tuberculosis* complex (MtbC), *M. leprae,* and mycobacteria other than MTBC and *M. leprae,* commonly known as *nontuberculous mycobacteria (NTM)* [1], [2]. The most well-known member of the MtbC is Mtb, an obligate human pathogen and the causative agent of *tuberculosis (TB)*, which is still one of the world's most serious public health issues [3], [4], [5]. The World Health Organization (WHO) has presented a worldwide TB report per year since 1997, which gives an up-to-date evaluation of the global TB status and analyzes progress and efforts in TB prevention, diagnosis, and treatment at the country, regional, and global levels [3], [6]. The 2020 Global TB Report was published on October 14, 2020, and was collected in the context of global TB control plans and United Nations (UN) objectives announced in the political declaration at the UN General Assembly high level conference on TB in New York in September 2018 [7]. Additionally, the 2021 Global Tuberculosis Report published by the World Health Organization showed that tuberculosis flared up again at the end of 2020, coinciding with the COVID-19 pandemic [8]. In 2019, an estimated 10.0 million persons had TB illness worldwide, with 1.2 million TB fatalities among HIV-negative people and 208, 000 deaths among HIV-positive people. Adults made up 88 percent of all TB patients, while children under the age of 15 made up 12 percent [7], [9], [10]. Tuberculosis is a treatable illness, and a lack of adequately cost-effective and reliable diagnostic techniques is a key impediment to accelerating worldwide TB burden reductions [11], [12]. Over the last decade, there has been a concentrated effort throughout the world to develop molecular screening procedures for tuberculosis, mostly by the detection of pathogen DNA, but also by targeting proteins or lipid compounds and the immunological response to TB infection. Early treatment and rendering patients non-infectious is critical to enhancing TB control and speeding up declining trend [11], [12]. Ultrasensitive new diagnostic procedures that could recognize pulmonary TB earlier at all points of healthcare would inform on treatment initiation, minimize the chances of transmission, improve treatment scanning and results, and inhibit long-term complications [13]. In this review, tuberculosis diagnostic biosensors were presented and discussed, in addition to investigating pathophysiology and ancient diagnostic methods. Given the number of studies conducted in the last decade, only studies from the last years (2018–2023) are presented in this study.

## 2 Pathophysiology of Mtb

*M. tuberculosis* is distributed by little airborne particles termed droplet nuclei that are produced by a patient with pulmonary or laryngeal TB via coughing, sneezing, talking, or singing. These microscopic particles can float in the air for minutes to hours [14]. The amount of bacilli in the droplets, the bacilli's virulence, the bacilli's exposure to ultraviolet (UV) radiation, the level of circulation, and the possibilities for aerosolization all have an effect on transmission [15]. The insertion of *M. tuberculosis* through into lungs causes respiratory system infection; although, the organisms might move to other tissues, like the lymphatics, pleura, bones/joints, or meninges, resulting in extra – pulmonary TB. When breathed, the infected droplets spread throughout the lungs. The bulk of the bacilli are caught in the upper portions of the lungs, where mucus-secreting goblet cells exist. The mucus generated traps foreign things, and the cilia on the surface of the cells regularly beat the mucus and its imprisoned particles upward for ejection [16]. This mechanism supplies the body with an immediate physical protection which inhibit the growth of bacteria in the majority of TB patients [17]. Bacteria in droplets that enter the alveoli without passing via the mucociliary pathway are rapidly encircled and consumed by alveolar macrophages [16], [18], the most numerous immune effector cells in alveolar spaces [19]. These macrophages, the body's second line of defense, are part of the innate immune system and allow the body to eliminate the intruding mycobacteria and avoid infection. Macrophages are easily available phagocytic cells that battle a wide range of diseases without the need for prior pathogen contact. Several processes and macrophage receptors are engaged in mycobacteria absorption [20]. Lipoarabinomannan from mycobacteria is a major substrate for a macrophage receptor [21]. The complement system is also involved in bacterial phagocytosis [22]. The C3 complement protein attaches to the cell wall and improves macrophage identification of mycobacteria. The C3 opsonization occurs quickly, even in the air passages of a host who has never been exposed to *M. tuberculosis* [23]. The following phagocytosis by macrophages sets off a chain of events that leads to either effective infection management, followed by latent TB, or progression to active illness, known as primary progressive tuberculosis. The effectiveness of the innate immunity and the ratio that happens between host defenses and the intruding pathogen largely affect the result [20], [24]. Mycobacteria continue to proliferate gradually after being swallowed by macrophages, with bacterial cell division happening every 25–32 h [18], [25]. Whether the infection is managed or advances, macrophages produce proteolytic enzymes and cytokines in an effort to destroy the bacteria [20], [26]. T lymphocytes, the cells that make up cell-mediated immunity, are drawn to the site by cytokines secretion. The development of granulomas surrounding *M. tuberculosis* organisms is the next protective step for those with full cell-mediated immunity [27]. These nodular-type lesions occur as a result of an aggregation of activated T lymphocytes and macrophages, which generates a micro – environment that inhibits mycobacteria proliferation and dissemination [28]. This environment kills macrophages and causes early solid necrosis in the lesion's core; although, the bacilli may adapt and evolve [29]. Furthermore, *M. tuberculosis* organisms may alter their phenotypic expression, such as protein modulation, to increase their chances of survival [22]. By two or three weeks, the necrotic environment simulates soft cheese and is characterized by low levels of oxygen, low pH, and restricted nutrients. This condition limits potential development and causes delay. Lesions in people with a healthy immune system often undergo fibrosis and calcification, successfully managing the infection and containing the bacilli in the inactive, recovered lesions [29]. In individuals with weakened immune systems, lesions proceed to primary progressive [22], [29].

## 3 Mtb related biomarkers

Antigen 85A (Ag85A), antigen 85B (Ag85B), heat shock protein 65 (Hsp65), early secretory target antigen (EAST-6) and heat shock protein X (HspX) are the antigens that have been researched and used specially to design a vaccine against Mtb. Mtb secretory and surface proteins (EAST-6) are the major antigens that confer immunity against tuberculosis[30]. These proteins are mycolyl transferase enzymes required for mycobacterial cell wall biosynthesis during tuberculosis pathogenesis [30]. In other words, these antigens can be used as critical biomarkers or biomolecules to detect Mbt infection. Accordingly, as reveal in the Table 2, these antigens have been widely used in biosensor technology research over the past decade.

**Table 1:** Conventional methods in detection of TB.

Methods	Disadvantages and limitation	Advantages/strengths	Ref
Serology	Inaccurate and highly inconsistent; WHO has recommended against their clinical use	Rapid, inexpensive, results within minutes, can be per- formed with minimal training	[1], [2]
Culture	Time-consuming, Need for advanced tool (biosafety practices (BSL-3)	Gold standard, Inexpensive	[1], [2]
TST	Not specific for *M. tuberculosis.* Does not discriminate latent infection from disease. May be positive with BCG vaccination/exposure to atypical mycobacteria. False negative in immunosuppressed, extra-pulmonary or miliary tuberculosis. Complications in test administration and explanation may lead to false results	Used for contact tracing, Preferred test in children, Relatively easy to perform, In expensive.	[3], [4]
Radiologic	Not specific for tuberculosis. Other infectious/grantdomatous/lymphoproliferative/occupational maladies can have showed similar patterns. Low sensitivity, especially for detection of lymphadenopathy. A normal result does not exclude ocular tuberculosis	Ability to monitor disease complications and treatment response, Higher sensitivity for detecting nodes Allows for surgical planning Characterization of lymph node morphology and enhancement May differentiate TB from non-TB lymphadenopathy.	[5], [6], [7]
IGRAs	Higher cost. Unavailable in most cases. Possibly more sensitive to detect latent infection than TST but does not discriminate it from disease. False in immunosuppressed states. Difficulties in collecting or transporting blood specimen may decrease the accuracy.	Independent of reader, reproducible, no booster effect, no interference with previous vaccinations	[8], [9], [10]
PCR	Limited availability and Higher cost. Variable sensitivity. Substandard sensitivity for non-respiratory specimens. Does not allow ruling out tuberculous etiology. Detects only DNA (more prone to contamination and microorganisms may not be viable or may be dormant)	Can distinguish Mtb from other acid-fast mycobacteria if smears are positive, Can detect Mtb and drug resistance	[10], [11], [12]

**Table 2:** Mtb developed biosensors (2018–2023).

Molecule	Platform	Technique	Matrix	Nanocomposite	Linear range	LOD	Ref
DNA sequences	Genosensor	CV, EIS, DPV	Clinical samples	Flower-like CNTs	1 fM–10 nM	0.33 fM	[13]
PNA	Electrochemical	EIS, CV, DPV	Clinical samples	TEMPO-NCC	1 × 10^−8^ M–1 × 10^−13^ M	3:14 × 10^−14^ M	[14]
DNA sequences	Aptasensor	CV	Serum samples	Pt@Au	1.0 × 10^−4^–2.0 × 10^2^ ng⋅mL^−1^	3.3 × 10^−5^ ng⋅mL^−1^	[15]
antigen ESAT-6	Genosensor	CV, SWV, DPV	Clinical samples	Au-nano-C_60_/NGS	10 fM–10 nM	3 fM	[16]
DNA sequences	Genosensor	CV	Sputum & Urine samples	Ag- nanoparticles	1 nM–100 nM (urine), 1 nM–100 nM (sputum)	16 fM	[17]
IS6110 gene	Fluorescent biosensor	FRET	Clinical sputum samples	QDs	0.05 nM–1.0 nM	35 pM	[18]
DNA sequences	Photothermal Biosensing	UV/Vis spectrometer	Genomic DNA & real samples	AuNP	4–1200 nM	0.28 nM	[19]
DNA sequences	Laser biosensor	EI, X-ray photoelectron spectroscopy	DNA samples	silver nanoparticles (AgNPs)	1 fM	10^−15^ M	[20]
CFP10-ESAT6 protein	Aptamer-Antibody sensor	DPV	Clinical sputum samples	Nano-labelled Fe_3_O_4_/Au MNPs	5–500 ng/mL	1.5 ng/mL	[21]
*IS6110* and *mtp40* genes	Lateral Flow Biosensor	mLAMP	Pure culture & clinical sputum samples	SA-PNPs	12.5 ng–125 fg	125 fg per vessel for the pure genomic DNA of Mtb, 4.8 × 10^3^ CFU/ml for the sputum samples	[22]
IS6110 gene	Genosensor	DPV	Clinical sputum samples	—	15 and 100 nM	4.4 nM	[23]
*IS6110* and *mpb64* genes	Genosensor	MCDA-LFB	Clinical sputum samples	LFBs based on nanoparticles	1 pg–100 fg	100 fg per reaction	[24]
MPT64 antigen	Aptasensor & immunosensor	ELONA, EIS, DPV	Clinical & real samples	AuNPs & C_60_NPs-N-CNTs/GO	1 fg/mL–1 ng/mL	0.33 fg/mL	[25]
ESAT-6 antigen	Aptasensor	SWV	Real samples	NG@Zr-MOF-on-Ce-MOF@Tb nanohybrid	100 fg mL^−1^–10 ng mL^−1^	12 fg mL^−1^	[26]
protein MPT64	Immunosensor	CV, EIS, DPV	Serum samples	Graphene oxide, Fe_3_O_4_ & PtNPs	5.0 fg·mL^−1^–1.0 ng·mL^−1^	0.34 fg·mL^−1^	[27]
DNA sequence	Genosensor	EIS	Clinical samples	Au/3DG	10 fM–0.1 µM	10 fM	[28]
Specific Oligonucleotide Sequence	Electrochemical	CV, EIS	Oligonucleotide	Poly(4-HPA)/GE	—	0.56 (±0.05) μM	[29]
Sputum	Genosensor	CV, DPV	Real sample	(MPA-Fe_3_O_4_	1.0 × 10^−6^–1.0 × 10^−12^ M	7.96 × 10^−13^ M	[30]
IS6110	Genosensor	CV, DPV	Clinical Samples	GO-CHI	7.86 pM–94.3 pM	3.4 pM	[31]
CFP10-ESAT6	Immunosensor	CV, DPV	Antigen (CFP10–ESAT6)	CdSe/ZnS QD/SiNP	40–100 ng/mL	1.5 × 10^−10^ g/mL	[32]
Ag85B antigen	Immunosensor	—	Spiked and real samples	SiQD & AuNRs	1 × 10^−3^–1 × 10^−10^ μg mL^−1^	13 pg mL^−1^	[33]
ESAT-6	Immunosensor	CV, EIS	Clinical samples	NiNPs-rGO	1–100 ng mL^−1^	1.042 ng mL^−1^	[34]
Mtb	Aptamer–Antibody Sandwich Assay	Chronoamperometry	Human serum sample & MT CFP10 antigen	Electrografted 4-carboxyphenyl diazonium salt	5–500 ng mL ^−1^	1.22 ng mL ^−1^ & 1.05 ng mL ^−1^ in human serum sample	[35]
*rpoB* gene	Genosensor	CV, EIS	Real samples	Fe_3_O_4_/polypyrrole nanocomposite	1 × 10^−6^–1 × 10^−12^ M	1 pM	[36]
H37Rv strain	Aptasensor	EIS	Clinical samples	AuNPs	1 × 10^2^ cfu/mL to, 1 × 10^7^ cfu/mL	100 cfu/mL	[37]
16S rDNA fragment	Genosensor	EIS	Sputum sample	AuNPs	1 × 10^2^–1 × 10^8^ CFU/mL	20 CFU/mL	[38]
DNA probe	Genosensor	Piezoelectric, EIS	Real sample	AuNPs	10^2^–10^8^ CFU mL^−1^	30 CFU mL^−1^	[39]
16S rDNA	Genosensor	EIS	Real sample	Ti_3_C_2_ MXenes & AuNPs	10^2^–10^8^ CFU mL^−1^	20 CFU mL^−1^	[40]
DNA sequences	Genosensor	CV, DPV	Clinical samples	MWCNT/PPy/KHApNps	100 pM–100 nM	50.3 pM	[41]
DNA sequences	Genosensor	DPV	Sputum clinical samples	HAPNPTs/PPY/MWCNTs	0.25–200.0 nM	0.141 nM	[42]
MPT6_4_	Aptasensor	DPV	Real sample	Au-nanoparticles	0.02–1000 pg·mL^−1^	10 fg·mL − 1	[43]
ESAT-6	Magnetoresistive biosensing chip	GMR technique	Real sample	Magnetic nanoparticles	—	12 pg/mL (∼2pM)	[44]
IS6110 gene	Genosensor	CV and EIS	Blood sample	Gold nanocrystals	0.1–1.0 × 10^5^ fM	0.031 fM	[45]
anti-CFP10-ESAT6	Immunosensor	DPV	Sputum samples	Fe_3_O_4_/Au MNPs	10–500 ng mL^−1^	1.5 ng mL^−1^	[46]
DNA sequences	Genosensor	LAMP	Blinded sputum samples	GPNP	—	1 pg	[47]
Heat shock protein X (HspX)	Immunosensor	SPR	Sputum Samples	—ـ	116–175 ng mL^−1^	0.63 ng mL^−1^	[48]
Mtb	Immunosensor	MACE	Sputum samples	GMNP	—	5000–10,000 CFU/mL	[49]
rAg85B	Biosensor & AMS	SPR	Clinical & Sera samples	—	6.25–200 μg/mL	—	[50]
IS*6110* and IS*1081*	Genosensor	SPMS-AIA	Clinical sputa	—	500 pg/µl–5 fg/µl	3.2 copies for IS6110 and 12 copies for IS1081 respectively	[51]

## 4 Mtb detection methods

Sputum is the frequently obtained clinical specimen from patients with pulmonary TB. Even though smear microscopy is a cost-effective and generally used method, its sensitivity was acceptable. Consequently, due to the need to expand the performance of current microbiological tests to provide rapid treatment, different approaches with varied sensitivity and specificity for TB diagnosis have been established. Here we debate the current techniques developed over the two past decades, as well as their strengths and weaknesses. Additionally, interferon gamma-based assays (IGRA) have been able to detect specific cellular immune responses to antigens expressed in *M. tuberculosis* (ESAT-6: Target early secretory antigen 6 and CFP-10: protein 10 of the culture filtrate), but not in BCG and in many environmental mycobacteria, would improve the sensitivity and specificity of tuberculosis diagnosis [31], [32]. Later, the WHO allotted a guideline not indorsing the use of such tests for the diagnosis of TB [33]. On the one hand, the only in vivo test existing to evaluate *M. tuberculosis* infection is the tuberculin skin test, which has suitable sensitivity but poor specificity [34]. On the other hand, the new interferon-gamma release assays are specific ex vivo tests [34]. Both techniques are based on the measurement of adaptive host immune response. But, none of these tests can precisely distinguish between active and latent TB [35]. Additional diagnostic tools have been advanced for the detection of M. tuberculosis, as well as viability and, drug susceptibility which can be assessed by metabolic activity responsiveness (recognition of mRNA synthesis or respiration), cell membrane integrity, or nucleic acid recognition [36], [37]. Along with these tests, conventional solid and new liquid media-based methods, which can obtain fast results, have been advanced; though, these tests are quite costly. Other methods (Table 1) have also been described for the detection of pathogenic mycobacteria [38]. Finding recommended, direct molecular detection of *M. tuberculosis* complex is specific and sensitive and PCR method should be used as an adjunct to other methods of laboratory diagnosis of TB [39]. Real-Time PCR assay to detect *M. tuberculosis* were employed for detection of TB in several studies [40], [41]. Some important and widely used methods in detection of TB were summarized in Table 1.

As revealed in Table 1, conventional methods have different disadvantages and limitations. To overcome these drawbacks, modern and sensitive techniques are developed extensively in recent years. Biosensors technology are one the most important methods.

## 5 Biosensors technology

Biosensors are analytical tools that combine biological materials like antibodies and nucleic acids with electronic systems to produce measurable signals [53]. Electronic devices sense, analyze, and send data regarding the existence of various chemical and physiological alterations, as well as biological compounds, in the environment [53]. Biosensors are available in a variety of forms and sizes and therefore can monitor and quantify low concentrations of biomarkers, particular infections, toxic substances, and pH values. Transducers, analytes, bioreceptors, electronic devices, and monitors are all examples of biosensors [53].

**Fig. 1:**
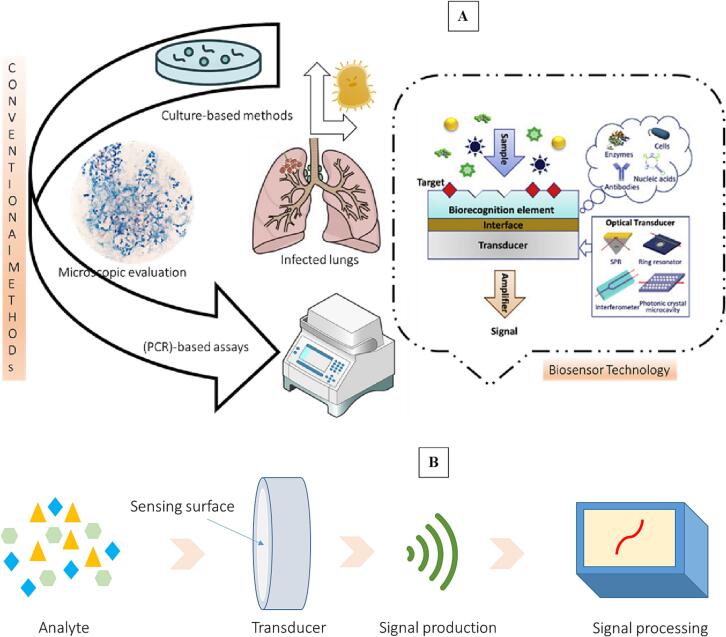
A) Trends in detection of Mtb, from conventional methods to nanotechnology-based methods B) Schematic illustration of biosensor technology.

The process of signaling creation during the contact between the bio-receptor and analyte is termed to as bio-recognition. Transducers are tools that change energy from one form into another [54], [55]. The transducer**,** is the essential component of a biosensor that translates the bio-recognition occurring into a detectable electrical signal when a chemical or biological target is present [54], [55]. Signalization refers to the process of converting energy. Transducers also provide electrical or optical signals that are proportional to the number of analyte-bioreceptor interactions. Transducers are categorized into three types based on their operating principles: electrochemical, optical, and mechanical [56], [57]. The transducer's electrical impulses are amplified and transformed to digital shape. The display unit quantifies the processed signals. A user interpretation device, such as a computer or a printer, generates the output such that the appropriate response is understandable and readable by the user [56], [57]. The trends in detection of Mtb, from conventional methods to nanotechnology-based methods and schematic illustration of biosensor technology were presented in the Fig. 1.

**Fig. 2:**
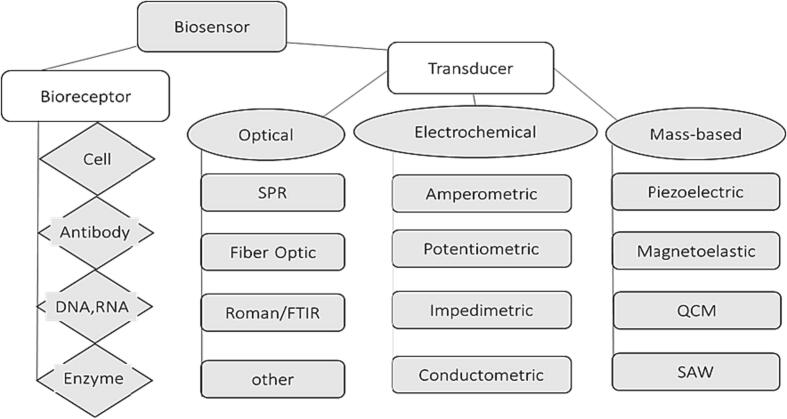
Biosensor classification.

Biosensors can be classified according to the mode of physicochemical transduction or the type of biorecognition element. Based on the transducer, biosensors can be classified as electrochemical, optical, thermal, and piezoelectric biosensors. Biosensor classification was illustrated in Fig. 2.

**Fig. 3:**
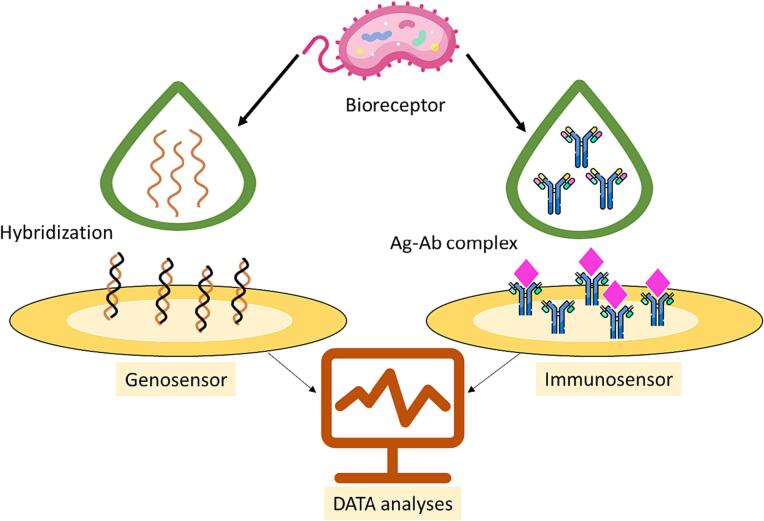
Schematic illustration of genosensor and immunosensor.

One of the main classifications related to biosensors is based on the type of biological receptor, among which genosensors and immunosensors are very important. Since most of the biosensors discussed in this study include genetic sensors and immunosensors, a description of these two types of diagnostic platforms will be discussed below [58], [59]. A gene is a distinct genomic region that contains the information necessary for protein synthesis. Gene-based identification methods, such as nucleotide-based sensors, have rapidly developed in recent years to detect genetic diseases, especially viral infections. Nucleotide-based detection “genesensors” are biological devices capable of recognizing a hybridization reaction based on a target nucleic acid (DNA or RNA) [59]. The single-stranded DNA (ssDNA) sequence is called the probe, and the target nucleic acid sequence is the recognition element of the gene-sensor. Their hybridization is monitored by direct profiling, but sometimes, the probe-target DNA complex present on the sensor surface may not induce the desired changes in the transduction values, thus, to improve detection limits, sandwich and competitive formats are preferred for direct use [60]. The principle of direct format is based on label-free detection by immobilizing the ssDNA probe on the surface of the probe, while in sandwich and competition format, the mixture of target DNA and ssDNA probe is incubated on the sensor surface is located through a specific marker used for identification [60], [61]. Immunosensors detect the specific immune response between an antibody and its target antigen, the formation of stable immune complexes, have been of interest in recent years as diagnostic tools which are applicable in industrial monitoring, clinical diagnostics, food monitoring, and environmental analysis [62], [63]. These devices are quick and simple to operate and are therefore suitable for point-of-care analysis. Electrochemical immunosensing is a well-known analytical technique that converts biological reactions into electrochemical reactions [62], [63]. The schematic illustration of genosensor and immunosensor are presented in Fig. 3.

## 6 Mtb biosensors

An amperometric DNA biosensor platform based on a flower-like carbon nanotubes-polyaniline nanohybrid and an enzyme-assisted signal amplification strategy was established for the precise and specific identification of TB in clinical samples. The developed electrode's electrochemical characteristics was studied using cyclic voltammetry (CV), differential pulse voltammetry (DPV) and impedance spectroscopy (EIS). A wide detection linear range of 1 fM–10 nM for Mtb target DNA was produced using a multiple signal amplification technique. More crucially, the universal DNA biosensor demonstrated good specificity and sensitivity for Mtb detection in clinical samples, suggesting that it might be a useful tool for Mtb testing as well as having tremendous promise for additional analytes [64].

An innovative peptide nuclide acid (PNA) electrochemical biosensor based on reduced graphene oxide (NH2-rGO)/2,2,6,6-tetramethylpiperidin-1-yl) oxyl nanocrystalline cellulose (TEMPO-NCC) for the detection of Mtb. The electrochemical characteristics of the proposed electrode was studied using CV and EIS. Meanwhile, DPV was used to assess the sensitivity and selectivity of the developed biosensor for detection of *M. tuberculosis* (DPV). Using methylene blue (MB) as the electrochemical indicator, the PNA probe-modified (NH2-rGO)/TEMPO-NCC response indicated that the constructed biosensor could differentiate between complementary, non-complementary and one-base mismatch DNA sequences. The designed electrochemical biosensor demonstrated a linear calibration curve in the concentration range of 1 × 10^−8^ M–1 × 10^−11^ M with a detection limit of 3.14 × 10^−14^ M. The created electrochemical biosensor was further tested using a polymerase chain reaction (PCR) product of *M. tuberculosis* DNA, which demonstrated successful outcomes in identifying between M. tuberculosis negative and positive samples [65].

An electrochemical genosensor was assembled to detect Mtb in lyophilized powder of oligonucleotides of *M. tuberculosis.* For this purpose, Methylene blue (MB), a photochemical indicator, was utilized to monitor the hybridization of target DNA using the differential pulse voltammetry (DPV) technique. Under ideal conditions, the detection range of the DNA biosensor was 10^−6^–10^−9^ M, with a detection limit of 7.853 10^−7^ M. The findings indicate that composite nanofibers have a high potential for use in a variety of DNA sensor applications [66].

**Fig. 4:**
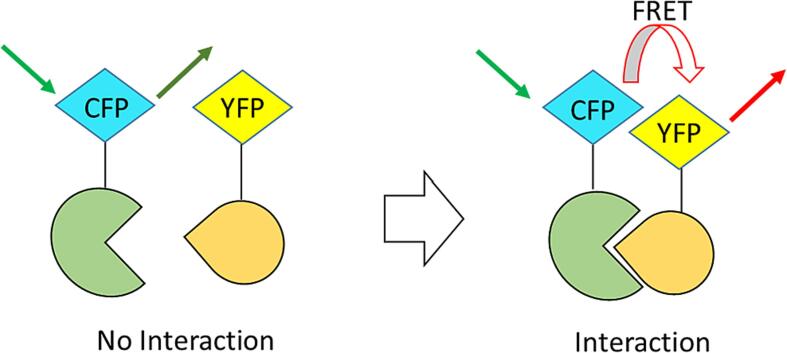
Schematic of FRET technique, Adapted from Ref. [71].

An electrochemical aptasensor was organized to detect *M. tuberculosis* in serum samples. This paper described a voltammetric aptasensor for ultrasensitive ESAT-6 detection. ESAT-6, a 6-kDa early secretory antigenic target, is an etiological agent released by Mtb. On a glassy carbon electrode, reduced graphene oxide doped with metal–organic framework (MOF-rGO) was deposited glassy carbon electrode (GCE). This promotes electroactive Toluidine Blue (TB) immobilization and enables electron transport from TB to the modified GCE. To further increase the reaction to TB, platinum/gold core/shell (Pt@Au) nanoparticles were employed to construct thiolated ESAT-6 binding aptamer (EBA) on a modified electrode. The modified GCE exhibits a linear response over a wide range from 1.0 × 10^−4^–2.0 × 10^2^ ng⋅mL^−1^ ESAT-6 and good sensitivity (LOD) for ESAT-6 as low as 3,3 × 10–5 ng⋅mL^−1^. The limit of detection (LOD) for ESAT-6 is as low as 3.3 × 10^−5^ ng⋅mL^−1^. When evaluating spiked human serum, it demonstrates acceptable specificity and repeatability [67]. For the detection of *M. tuberculosis,* a novel electrochemical genosensor was designed. For the first time, a nanohybrid composed of gold nanoparticles decorated with nitrogen-doped fullerene nanoparticles/graphene sheets (Au-nano-C60/NGS) effectively provided a new signaling marker for generating a signal response without adding redox molecules and is then labeled with a signal probe (SP) to establish a tracking label to achieve signal amplification. A biotin-avidin method was also used to immobilize numerous capture probes (CPs), which increased the sensitivity of the suggested biosensor. To stimulate the inherent redox activity of the tracer label, the suggested electrochemical DNA biosensor was incubated with tetraoctylammonium bromide (TOAB), which was utilized as an accelerator, leading in a differentiating current response. The suggested electrochemical DNA biosensor has a wide linear range for Mtb determination ranging from 10 fM to 10 nM and a LOD of 3 fM. Furthermore, proposed biosensor identifies Mtb from other pathogenic pathogens as well as mismatched DNA sequences. More notably, it has been used in clinical detection and has demonstrated an exceptional capacity to recognize the Mtb in clinical samples. This discovered approach has a high potential for application in the early diagnosis and monitoring of tuberculosis [68]. An electrochemical biosensor was created for highly specific DNA insertion element 6110 (IS6110) detection of Mtb. This biosensor constructed based on a PCR amplified DNA product on the surface of the working electrode built on FTO-Glass. In this work CV is performed with an Ag/AgCl reference electrode and a platinum counter electrode [69]. Förster resonance energy transfer (FRET) is a procedure involving the non-radiative transfer of energy from a 'donor' fluorophore to an 'acceptor' fluorophore [70]. Briefly, FRET is a distance-dependent interaction between pairs of closely spaced fluorescent donors and acceptors, in which fluorescence energy is radiatively transferred from an excited donor to the corresponding acceptor molecule, Fig. 4 [71]. The efficiency of FRET strongly depends on the distance between the donor and acceptor and the spectral overlap between donor emission and acceptor excitation.

A new optical biosensor based on the FRET method, proposed as a universal fluorescent biosensor for detecting of Mtb unique insertion sequence *IS6110* gene fragment [72]. This approach demonstrated good sensitivity, specificity, and excellent potential for rapid TB detection [72]. For the first time, a simple, low-cost, and ubiquitous gold nanoparticle (AuNP) aggregation-induced photothermal biosensing platform has been designed and used for optical quantitative genetic detection utilizing a typical thermometer. Visual quantitative biological analysis may be accomplished by simply capturing temperature signals with a simple thermometer and utilizing the photothermal effect of target-induced gold nanoparticle aggregation. When compared to traditional genetic testing procedures, it is label- and amplification-free and may be done in 40 min without the use of any sophisticated analytical tools. To demonstrate the use of this photothermal biosensing technology, DNA from Mtb was employed as a model target. Despite the lack of a pricey equipment, good sensitivity and specificity were obtained, with a LOD of 0.28 nM, which was approximately 10-fold lower than the colorimetric approach employing a spectrometer. This AuNP aggregation-induced photothermal biosensing technique supplies a simple, low-cost, and ubiquitous platform for wide visual quantitative detection of nucleic acids and many other biomolecules, especially in point-of-care (POC) biosensing applications [73]. Researchers created a green graphene nanofiber laser biosensor (LSG-NF) decorated with oil palm lignin-based synthetic silver nanoparticles. To validate the sensing efficiency, a selective DNA sample captured on AgNPs was examined for specific binding with Mtb target DNA using selective hybridization and mismatch analysis. Electrochemical impedance experiments revealed acceptable sensitive detection of up to 1 fM, with a detection limit of 10^−15^ M calculated by assuming the signal-to-noise ratio (S/N = 3:1) as 3 σ. The identification of phosphorus and nitrogen peaks using X-ray photoelectron spectroscopy and Fourier-transform infrared spectroscopy demonstrated successful DNA immobilization and hybridization. The planned system showed excellent stability and repeatability. This technique provides a cost-effective potential sensing system for the determination of *M. tuberculosis* [74].

A portable electrochemical aptamer-antibody based sandwich biosensor has been planned and productively industrialized using an aptamer bioreceptor immobilized onto a SPE surface for Mtb detection in clinical sputum samples. In the sensing approach, a CFP10-ESAT6 binding aptamer was immobilized onto a graphene/polyaniline (GP/PANI)-modified gold working electrode by covalent binding via glutaraldehyde linkage [75]. Nanoparticle-based lateral flow biosensor coupled with a multiplex loop-mediated isothermal amplification was developed for fast and optical distinction of Mtb from the other Mtb complex. Planned system showed acceptable sensitivity and it can be used as a potential screening tool for TB in clinical, field, and basic laboratory settings [76]. Lateral flow biosensor with two target genes based multiple cross displacement amplification combined with a for the detection of *M. tuberculosis* complex was advanced. The MCDA-LFB assay aiming the *IS6110* and *mpb64* genes was a simple, fast, sensitive and reliable detection technique, and it has potential significance for the screening and treatment of TB [77].

**Fig. 5:**
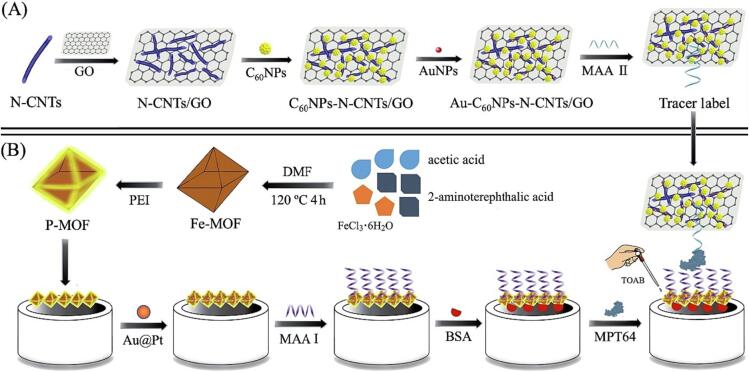
Developed aptasensor for detection of Mtb, with permission from Ref. [78].

A sandwich-type electrochemical aptasensor, Fig. 5, for Mtb, MPT64 antigen discovery using C60NPs decorated N-CNTs/GO nanocomposite coupled with conductive PEI-functionalized metal–organic framework was engineered. Planned biosensor displayed a wide linear range and acceptable LOD. Additionally, developed system can be detecting MPT64 antigen in human serum, demonstrating a favorable outlook for TB diagnosis in clinical practice [78].

**Fig. 6:**
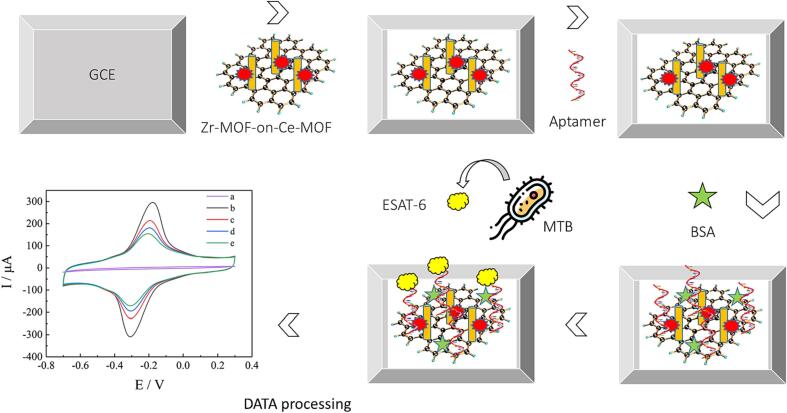
Developed electrochemical aptasensor, adapted from Ref. [79].

A label-free electrochemical aptasensor, Fig. 6, is described for ultra-sensitive detection of the 6-kDa early secreted antigenic target (ESAT-6) as one of the most important TB antigen. The bimetallic organic framework (b-MOF) of Zr-MOF-On-Ce-MOF was ornamented with nitrogen-doped graphene (NG) and used as the matrix for electroactive toluidine blue (Tb) to form the nanocomposite. The organized nanohybrid with exceptional hydrophilicity, dispersibility, and large specific surface showed noteworthy electrochemical response [79].

**Fig. 7:**
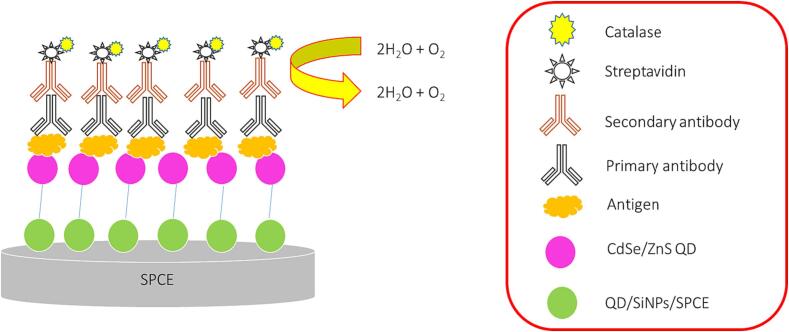
CdSe/ZnS QD/SiNP Electrochemical Immunosensor for the Detection of Mtb, adapted from Ref. [83].

An electrochemical immunoassay for high sensitive determination of the Mtb secretory protein MPT64 which is an antigen for early diagnosis of infection with Mtb. The protein G was used to immobilize antibodies against MPT64 on a gold electrode. Additionally, nanocomposite of type GO@Fe_3_O_4_@Pt was applied as a signal reporter with exceptional recyclability and catalytic activity [80]. An electrochemical genosensor was advanced for detection of the genetic codes preserved from the Mtb, its specific complementary target and interfering with more than 60 % of complementarity [81]. An ultra-sensitive electrochemical genosensor based on nanocellulose crystalline functionalized cetyl trimethyl ammonium bromide (NCC/CTAB) with functionalized iron oxide mercaptopropionic acid (MPA-Fe_3_O_4_) nanoparticle and has been fabricated for the detection of Mtb. In this research, a simple drop cast method was used to deposit solution of MPA-Fe_3_O_4_/NCC/CTAB onto the surface of the screen printed carbon electrode (SPCE) [82]. A simple electrochemical-based SPCE biosensor Fig. 7, for Mtb detection, using CFP10-ESAT6 as protein biomarker. In this work the active surface area of CdSe/ZnS QD/SiNPs/SPCE was applied as a positive surface that showed wide linearity and acceptable LOD [83].

A simple and sensitive sandwich assay for recognition of Mtb, Ag85B antigen using quantum dots and gold nanorods was engineered. A genetically planned recombinant antibody (GBP-50B14 and SiBP-8B3) was bound to surfaces of AuNRs and SiQDs respectively, without any surface modification. Created biosensor displayed a good sensitivity and selectivity for Ag85B-expressing Mtb detection [84].

A stable and recyclable immunosensor for the fast detection of Mtb based on the detection and quantification of ESAT-6 by CV. The immunosensor was synthesized by polymerizing aniline dispersed with the rGO and NiNPs, followed by surface modification of the electroconductive polyaniline (PANI) film with anti-ESAT-6 antibody. Physicochemical description of the prepared materials was performed by several analytical techniques appropriately [85]. An ecofriendly aptamer-antibody-based sandwich biosensor employing ChA for rapid and primary detection of Mtb CFP10 antigen was described. The recommended aptamer-based sensor was simple, sensitive, and disposable, thus suitable for point-of-care tuberculosis detection. This work similarly, highlights the use of CFP10 aptamer as a cost-effective and stable alternative reagent to antibodies for the progress of an improved identification of tuberculosis [86]. An innovaitive biosensor for the fast detection and differentiation of Mtb was proposed properly. For this purpose, multiplex loop-mediated isothermal amplification (mLAMP) combined with a nanoparticle-based lateral flow biosensor (LFB), was established (mLAMP-LFB) were employed [76]. Similarly, for sensitive detection of *IS6110* gene as one of the important Mtb biomarker. Electrochemical analyzes were performed using DPV by measuring the methylene blue reduction signal after and before hybridization between the synthetic target and the probe or between DNA extracted from clinical sputum samples [87]. Gold nanorods integrate a novel 3D graphene nanocomposite for selective biosensing for rapid recognition of Mtb. Developed genosensor proved high-performance bio-sensing and opens a novel opportunity for Mtb detection [88]. A biosensor method based-on poly(4-HPA)/GE platform efficiency was fabricated for detection Mtb. The polymer used facilitates target hybridization and optimizes probe adsorption parameters, leading to progress in reducing the methylene blue reduction signal [81]. Iron Oxide/Nanocellulose Crystalline nanocomposite was fabricated as a genosensor based on modified SPCE for determination of Mtb. Wide range linearity and good LOD was reported for real samples analyses [82]. Graphene oxide-chitosan nanocomposite (GO-CHI), as a biocompatible matrix, was immobilized on the ITO surface to form an active functional electrochemical sensor for Mtb detection. A DNA probe, specific for IS6110, which electrostatically fixed on a positively charged electrode surface were employed for sensitive and specific detection of Mtb by CV and DPV [89]. An original immunesensor based on surface enhanced CdSe/ZnS QD/SiNP platform was assembly for sensitive detection of Mtb CFP10-ESAT6 antigens. Developed biosensor displayed good reproducibility of target analyte with a qualified standard deviation [90]. E-DNA of *rpoB* gene as an important resistance gene was determined in real samples using Fe_3_O_4_/polypyrrole nanocomposite. The resulting biosensor can detect the *rpoB* gene in PCR-amplified genomic DNA samples and can also differentiate between the wild-type *rpoB* gene and a single-nucleotide mutated *rpoB* gene that confers resistance to rifampicin [91]. Additionally, the sensor can selectively detect wild-type and mutant DNA in genomic samples without the need for PCR amplification [91]. AuNPs-DNA combined with Aptamer were fabricated for detection of strain H37Rv Mtb. The planned sensor was sensitive and specific and rapid operation [92]. An original 16S rDNA series piezoelectric quartz crystal (MSPQC) sensor based on Exonuclease III (Exo III)-assisted target recycling was improved for Mtb recognition. The Mtb-specific 16S rDNA fragment was used as a biomarker, and DNA capture probes complementary to the biomarker were designed and modified on the AuNP surface. Exo III, was capable of recognizing hybrid duplexes and selectively processing the DNA capture probe, which used to facilitate the digestion cycle by digesting the DNA capture probe and discharging the target fragment [93]. A piezoelectric sensing method based on AuNPs-mediated enzyme assisted signal amplification platform was developed for detection of Mtb 16 S rDNA fragment. The created sensor can enable rapid and sensitive detection of *M. tuberculosis.* Furthermore, this method can be converted to various microbial targets, which is suitable for the further development of small handheld devices and multifunctional detection [94]. A sensor platform based-on two-dimensional Ti_3_C_2_ Mxenes 16S rDNA gene was advanced for electrochemicaly sensing of Mtb. The planned biosystem was applicable for rapid detection of specific fragment of 16S rDNA of Mtb H37Ra strain [95]. A novel nanobiosensor as a rapid, inexpensive method was advanced for Mtb detection. The engineered sensor was PCR-free with high specificity and sensitivity, using Multi walled carbon nanotubes (MWCNTs), potassium-substituted hydroxyapatite (KHAp) nanoparticles and polypyrrole [96]. A new electrochemical genosensor was developed based on HAPNPTs/PPY/MWCNTs nanocomposite for detecting Mtb. Assembled nanocomposite surface hybridized to a complementary target sequence based on the oxidation signal of the electroactive methylene blue on the surface of the modified GCE using DPV method [97]. A high sensetive aptasensor was designated for the CV determination of the Mtb antigen MPT64 in human serum. Initially, amino-modified Zr(IV)-based metal–organic frameworks with high specific surface area were produced and used as supports for gold nanoparticles and aptamers [98]. Magnetoresistance (GMR) sensor was developed for ESAT-6 detection. The produced tool shows that ESAT-6 concentrations can be detected in the pg/mL range compared to other transduction techniques available for ESAT-6 detection, and furthermore, the signal intensity increases with increased concentration [99]. Gold nanocrystals were used for electrochemical detection of the Mtb IS6110 gene. The detection signal was further enhanced by the catalyzed redox reaction of thionine with gold nanocrystals. The DPV signal increased with increasing target DNA concentration within range and with good LOD. This method provides extremely high sensitivity, specificity, and stability and has been successfully applied to detect tuberculosis in human blood [100]. A cost-effective, rapid, and portable, sandwich immunosensor approach was settled to detect Mtb in sputum samples. Using a sandwich-type immunosensor, with immobilization of anti-CFP10-ESAT6 antibody on a graphene/polyaniline (GP/PANI) modified SPE. After incubation with the target antigen CFP10-ESAT6, iron/gold magnetic nanoparticles (Fe_3_O_4_/Au MNPs) conjugated with anti-CFP10-ESAT6 antibody were applied to complete the sandwich format [101]. Combined ring-mediated isothermal amplification using a graphene-based electrochemical gene sensor was improved for the real-time identification of Mtb-specific DNA amplicons. The technique developed is a highly specific technique that detects the presence of tuberculosis in all sputum samples with the highest accuracy. Furthermore, this method can be easily applied clinically due to its affordability, speed, and feasibility without the need for advanced tools [102]. A facile, portable, and inexpensive biosensor was proposed to detect heat shock protein X (HspX) of Mtb. Following this approach, established a label-free Surface plasmon resonance (SPR) biosensor for direct immunoassay and quantification of X (HspX) as a well-established biomarker of pathogens. This method is based on highly specific monoclonal antibodies that have been previously immobilized on the plasmonic sensor surface [103]. A novel nanoparticle-based biological colorimetric assay (NCBA) for the detection of tuberculosis that is globally accessible and inexpensive with culture-equivalent sensitivity has been constructed. The results showed that NCBA had high sensitivity and specificity, respectively, compared to the control culture method [104]. Mass spectrometry (MS) is a method of analyzing target complexes based on their mass/charge ratio. MS separates and senses the composition of components by the difference in mass of atoms, molecules or molecular fragments of that substance thanks to the principle that charged particles have the ability to deflect in an electromagnetic field [105]. Determination of epitope and affinity of recombinant Mtb Ag85B antigen for anti-Ag85 antibody by proteolytic affinity mass spectrometry and biosensor analysis. These combined methods allowed the recognition of different epitope regions clustered on recombinant Mtb antigens, and their affinity binding constants when interacting with specific antibodies, and showed the importance of protecting against excessive glycosylation [106]. A silicon ring photonic sensor and asymmetric isothermal amplification technique (SPMS-AIA) was developed for rapid, isothermal, label-free, and real-time detection of Mtb. The performance of the SPMS-AIA platform was evaluated by detecting two IS6110 and IS1081 as MtbC-specific biomarkers [107]. More detail of Mtb developed biosensors (2018–2023) were summarized in the Table 2.

## 7 Conclusion

Biosensors have attracted much attention for *M. tuberculosis* due to their sensitivity and reduced assay time. Several biosensors have been established, including electrochemical, SPR, optical, mechanical, and QCM. The inherent specificity and sensitivity of developed biosensors make them ideal candidates for clinical applications. In developed biosensors, *M. tuberculosis* have been used as biorecognition elements in various protocols, such as electrode surface creation and immobilization on nanomaterials. Thus, the inherent properties of bacteria to differentially bind to specific analytes characterizes nanoparticle as interesting building blocks for new interdisciplinary electrochemical studies. Therefore, linking highly specific biosensors by an electrochemical method is a highly sensitive, inexpensive, simple and promising way to obtain superior sensors compared to other detection approaches. Although biosensors are described as sensitive, easy-to-use and cost-effective tools, their development still faces many challenges. In other words, although much progress has been made in the field of biosensors for microbial detection, there is still a need for modern biosensors to overcome the limitations of developed biosensors. It should be noted that currently most of the biosensors developed are used in research and in most cases their miniaturization and commercialization have not yet been achieved. Therefore, future studies should focus on the practical use of biosensors in medical diagnostic centers as an alternative tool to old methods. According to the advances achieved in the field of biosensing, the realization of an ideal detection system can be expected in the near future.

**Ethics (NA)**

**Consent for publication (NA)**

**Availability of data and materials (NA)**

**Funding (NA)**

## CRediT authorship contribution statement

**Ahmad Mobed:** Conceptualization, Supervision. **Mohammad Darvishi:** Writing – original draft. **Fereshteh Kohansal:** Data curation. **Fatemeh Moradi Dehfooli:** Conceptualization, Supervision. **Iraj Alipourfard:** Investigation. **Amir Tahavvori:** Writing – original draft. **Farhood Ghazi:** Writing – original draft.

## Declaration of competing interest

The authors declare that they have no known competing financial interests or personal relationships that could have appeared to influence the work reported in this paper.
